# Defective B cell ontogeny and humoral immune response in mice prematurely expressing human complement receptor 2 (CR2, CD21) is similar to that seen in aging wild type mice

**DOI:** 10.1016/j.molimm.2009.03.007

**Published:** 2009-06

**Authors:** Jason P. Twohig, Isabel Y. Pappworth, Baalasubramanian Sivasankar, Liudmila Kulik, Melanie Bull, V. Michael Holers, Eddie C.Y. Wang, Kevin J. Marchbank

**Affiliations:** a Institute of Human Genetics, Newcastle University, Center for Life, Central Parkway, Newcastle NE1 3BZ, UK; b Department of Medical Biochemistry and Immunology, School of Medicine, Cardiff University, Cardiff, UK; c Departments of Medicine and Immunology, University of Colorado, SOM, Denver, CO, USA

**Keywords:** Transgenic/knockout, Complement, B lymphocytes

## Abstract

Mice prematurely expressing human CR2 (hCR2) in the B cell lineage have a defective B cell ontogeny and humoral immune response. We have previously determined altered tyrosine phosphorylation patterns within hCR2 transgenic mice, suggesting that irreversible changes in B cell signaling pathways had occurred, which could explain the B cell unresponsiveness associated with hCR2 transgene expression. In support of that assertion, we found that increasing antigen dose or addition of adjuvant had a minimal impact on the ability of B cells to respond to antigen. However, analysis of aged hCR2^high^ mice (1 year plus) revealed that both B cell numbers, B cell sub-population distribution including expansion of a newly described B regulatory cell subset, and immune responses were comparable with age-matched hCR2 negative mice. Finally, we established that B cell unresponsiveness to antigen in aging wild type mice (1 year plus) was equivalent to that noted in 3-month-old hCR2^high^ mice. This data provides evidence that 3-month-old hCR2^high^ mice have a humoral immune system resembling aged mice and suggests that further examination of the precise molecular and cellular parallells between aged wild type mice and 3-month-old hCR2^high^ mice could provide an important insight into the mechanisms which lead to B cell unresponsiveness in the aging immune system.

## 1 Introduction

Aging negatively impacts the immune system at many levels and results in a decreased responsiveness to infectious agents and vaccination (Johnson and Cambier, 2004). Declining antibody responses with age were initially suggested to result from cellular deficiency (Schwab et al., 1989). However, the total number of B cells or Ig-secreting B cells remains similar between young and aging individuals (Johnson et al., 2002a,b; Zhao et al., 1995). This has been rationalized by the idea that immune dysregulation or remodeling is a more likely reason for the lack of response to antigens in aging individuals (Bovbjerg et al., 1991; Franceschi et al., 1995) and is supported by several age-related changes in key immune cells, including loss of T helper cell function (Krogsrud and Perkins, 1977; Miller, 1996; Yang et al., 1996) as well as greater production of low affinity antibodies (Doria et al., 1978; Goidl et al., 1976), increased incidence of autoreactive antibodies (Hayashi et al., 1989), different V_H_ usage (Nicoletti et al., 1991; Riley et al., 1989) and an increased life span of aged B cells (Kline et al., 1999). In short, this has led to the view that the decrease in antibody responses to foreign antigen in aged individuals is not due to a decrease in humoral immunity *per se* but a change in the overall B-cell repertoire, which directly influences the quantity and quality of the antibody produced (Weksler, 2000).

Over the last decade, we have been examining the humoral immune response in human complement receptor 2 (hCR2) transgenic (tg) mice (Birrell et al., 2005; Kulik et al., 2007; Marchbank et al., 2000, 2002). The role for CR2 in the development and maintenance of the humoral response to complement opsonised T-dependent (TD) antigens (Ags) was first discovered by several *in vivo* studies using CR1/2 blocking Abs and a study using CR2–IgG fusion protein (Gustavsson et al., 1995; Hebell et al., 1991; Heyman et al., 1990; Thyphronitis et al., 1991). This role was confirmed and expanded by the independent generation, by gene targeting, of 3 lines of *Cr2*^−/−^ mice (Ahearn et al., 1996; Haas et al., 2002; Molina et al., 1996). *Cr2*^−/−^ mice have been shown to have a defect in response to T-independent (TI) Ag (Haas et al., 2002) and to T-dependent antigens (Ahearn et al., 1996; Molina et al., 1996) as well as many other facets of the humoral immune response and clearly demonstrated that CR2 is central to the breadth of B cell function (reviewed Holers, 2005).

To date and from our point of view our hCR2^high^ mice, which express hCR2 under the control of a lambda promoter/enhancer minigene, have yielded the most interesting data (Birrell et al., 2005; Kulik et al., 2007; Marchbank et al., 2002; Twohig et al., 2007; Pappworth et al., 2009). In contrast to mice expressing hCR2 under its own promoter (Marchbank et al., 2000) or endogenous mouse CR2 (Takahashi et al., 1997; Tedder et al., 1984), expression of hCR2 was detected during the early stages of B cell development and not restricted to transitional and mature B cells. This ‘early’ expression of CR2 apparently results in a 40–60% reduction in mature B cell numbers, decreased serum IgG and failed humoral immune response, particularly evident in the germinal center reaction (Birrell et al., 2005; Marchbank et al., 2002). These changes occur despite association of hCR2 with mouse CD19 and amplified Ca^2+^ flux after CR2/BCR cross-linking, which are reliable read outs of co-receptor activity on the B cell (Bradbury et al., 1992; Fearon and Carter, 1995; Matsumoto et al., 1991; Tedder et al., 1994). Further examination of the CR2 signaling uncovered evidence of alteration in tyrosine phosphorylation patterns in response to BCR and CR2/CD19 cross-linking in the hCR2^high^ mice.

Importantly as a likely result of these changes, hCR2^high^ mice are almost completely protected from the onset of an organ specific autoimmune disease (Kulik et al., 2007). Interestingly, hCR2^high^ B6^lpr^ mice were also partially protected from systemic autoimmune disease and exhibited a modified B-cell repertoire (Pappworth et al., 2009). Thus, the defects noted in the hCR2^high^ mice appear largely consistent with the concept of CR2 and complement playing a central role in the elimination of self-reactive B cells and maintenance of B cell tolerance (Carroll, 2000; Prodeus et al., 1998).

Attempts to reverse the phenotype displayed by hCR2^high^ B cells through backcrossing onto the CD19^−/−^ or C3^−/−^ backgrounds (in an endeavor to remove the signaling associated with these molecules) had little restorative effect (Twohig et al., 2007). However, data from these studies did confirm that hCR2 was integrated into murine B cell signaling during the development of the mouse B cell in the bone marrow, such that absence of C3 resulted in a 3-fold increase in surface expression of hCR2 and the presence of CD19 was found to be vital for survival of developing B cells which also expressed hCR2.

The difference in the level of protection noted between the collagen induced arthritis study (Kulik et al., 2007) and the recent B6.lpr study (Pappworth et al., 2009) led us to question the irreversible nature of the alteration in B cell functional status in C57Bl/6 (B6) mice. Analysis of anti-nuclear antigen (ANA) levels in B6 and hCR2^high^ mice over time revealed that whilst mice expressing hCR2 develop significantly less ANA at 3 months of age, the levels of ANA increase to similar levels to that seen in wild type littermates by 12 months of age. This data inferred that B cell function was possibly reversible given the right stimulus. However, we found that increased antigen dose failed to overcome the loss of the germinal center reaction in hCR2^high^ mice, although some recovery in total Ig levels was noted. Analysis of aging hCR2^high^ mice indicated that there was a partial recovery in immune response that was linked to a re-adjustment of B cell sub-populations in the spleen. Examination of wild type immune response to a TD antigen, sheep red blood cells (SRBC), as mice aged confirmed that germinal center responses almost entirely fail in B6 mice over time and that marked changes occur in the distribution of cells in each of the B cell sub-populations in the spleen. On the whole, these changes were similar to that seen in young hCR2^high^ mice. Thus, we have concluded that the hCR2^high^ mice appeared to have adopted a B cell phenotype at 3 months of age that is highly reminiscent of that seen in much older wild type mice.

## 2 Materials and methods

### 2.1 Cells

Peripheral blood lymphocytes (PBL) from mice were collected into 20 μl of heparin via a tail bleed and washed once in cold PBS. Bone marrow B cells were collected by flushing mouse femurs with cold PBS. Isolated spleens were ground into single cell suspensions using frosted glass slides and transferred to 15 ml conical tubes on ice. Large debris settled after a 10 min incubation and the supernatant was transferred to a new tube. Cells were pelleted and washed once with staining buffer (PBS, 1% FBS, 0.02% sodium azide). All mouse derived samples were incubated with 0.5–1 ml of ammonium chloride red blood cell (RBC) lysis buffer and incubated at room temperature for 1–2 min. The cells were then washed with 1 ml staining buffer 1–2 times. Cells were then counted and (1–3) × 10^6^ cells/ml used per analysis. Cells were then stained as described below.

### 2.2 Antibodies

Purified and biotin conjugated 171 (anti-hCR2), purified and biotin–IgG_1_ (isotype control) were produced in the laboratory following standard methods. Purified 2.4G2 (anti-mCD16/mCD32, FcBlock), phycoerythrin (PE) conjugated B-Ly-4 (anti-hCR2), FITC conjugated anti-GL-7 (Ly-77); biotin conjugated 145-2C11 (anti-CD3ɛ), FITC or PerCP conjugated RA3-6B2 (anti-mCD45R, B220), PE conjugated S7 (anti-CD43, Ly-48, leukosialin), biotin conjugated (anti-CD24, heat stable antigen), PerCP conjugated CD5 (Ly-1), PE conjugated anti-CD1d (CD1.1, Ly-38); anti-CD23 (anti-FcɛIII) and streptavidin (SA)–allophycocynin (APC) were all obtained from BD Pharmingen (Oxford, UK). Biotin anti-CD11b (MAC-1 α chain) was obtained from Invitrogen (Paisley, UK).

### 2.3 Mice

All mice in this study are on the C57Bl/6 (B6) genetic background and additionally have the transgenes and genetic deletions described. The lambda human CR2 transgenic mice (hCR2^high^) used in this study were generated and screened by PCR and/or flow cytometry as previously described (Marchbank et al., 2002, 2000). Additional mice used are the *Cr2*^−/−^ mice (confirmed by PCR or flow cytometry using biotinylated 7E9), the C3^−/−^ mice (screened by ELISA or Western blot as previously described (Twohig et al., 2007)). All mice used were age and sex matched littermates from in house colonies maintained at Cardiff University.

### 2.4 Flow cytometry

After RBC lysis, cells were washed and then resuspended in 10 μg/ml of 2.4G2 antibody in order to block Fc receptors. After 15 min incubation on ice cells were washed in staining buffer. Cells were resuspended in 100 μl staining buffer containing primary Ab (0.1–3 μg/ml) and 1 μl anti-B220-FITC, where appropriate. Cells were incubated for 30 min on ice in the dark. After incubation, cells were washed in staining buffer 3 times and then incubated with the appropriate streptavidin conjugated fluorochrome to detect biotin labeled primary Abs. Following incubation, cells were washed as above and then resuspended in staining buffer containing 1% formaldehyde. Flow cytometry was carried using a FACSCalibur (BD, Oxford, UK).

### 2.5 Sheep red blood cells (SRBC) immunizations and serum immunoglobulin assays

SRBC (TCS Bioscience, Buckinghamshire, UK) were washed 3 times and resuspended to the required concentration in PBS. Mice were injected intraperitoneally with 500 μl of SRBC in PBS at day 0 or subcutaneously with 100 μl SRBC mixed 1:1 with complete Freunds adjuvant injected as required. Mice were boosted with 100 μl SRBC in incomplete Freunds adjuvant at D28 as required. Serum was collected at days 0, 7, 14, 21, 28 and 35 during time courses. For germinal center analysis, mice were immunized on day 0 and serum collected at day 10. Detection of Ab to SRBC was carried out using flow cytometry. SRBC were washed 3 times in PBS and resuspended at a 0.05% (v/v) solution in flow buffer. 100 μl of SRBC solution was plated on ELISA plates and heat inactivated (56 °C for 30 min) mouse anti-sera added. Standard curves were generated with doubly diluted control anti-sera to confirm that test samples fell within the linear range of the assay for each isotype used. Test samples were incubated in triplicate at room temperature for 30 min and then washed 3 times in flow buffer. Plates were then incubated with 1/250 dilution of anti-mouse IgM–APC or anti-mouse IgG–PE (Jackson labs) for 30 min at room temperature. SRBC were washed 3 times and then immediately analysed. Between 4 and 6 mice were used in each group as stated.

### 2.6 Immunohistochemisty

Mice were injected with SRBC by the i.p. route as described above, and spleens were collected on day 10. Spleens were mounted in OCT compound (EMS Laboratories) and snap frozen in isopentane. Frozen sections (10 μm) were cut on a CME cryostat (Thermo Shandon) and dried at 37 °C for 30 min before being fixed for 10 min in acetone. After a brief wash in PBS, endogenous peroxidase activity was quenched by incubating the section with 0.1% H_2_O_2_ in PBS for 20 min. Biotin and avidin binding sites were then blocked using a biotin/avidin blocking kit (Vector Laboratories). Sections were incubated with 1:100 rat anti-mouse IgD (BD Pharmingen) and 1:300 biotin conjugated PNA (Vector Laboratories) in 1% BSA/PBS for 1 h at room temperature. Slides were washed 3 times with PBS. Sections were then incubated with 1:300 rabbit anti-rat HRP (Stratech) and 1:400 Extra-avidin-AP (Sigma–Aldrich) for 1 h at room temperature. Slides were then washed 3 times in PBS. The sections were then developed using a BCIP/NBT alkaline phosphatase kit (Vector Laboratories), according to the manufacturer's instructions, and after a brief wash in PBS, HRP was developed using 0.05% diaminobenzidine/0.02% H_2_O_2_. Slides were rinsed with tap water after development, dehydrated, and mounted using Surgipath (Bretton) mounting medium. Sections were viewed using a Leica microscope with attached digital camera and analysed using Improvision Openlab software (Improvision).

### 2.7 Anti-nuclear antigen ELISA

Blood was collected from age- and sex matched wild type B6 or B6.hCR2^high^ mice by tail vein nick and allowed to clot. Sera were then applied to a quantitative ANA ELISA as directed by the manufacturer's instructions (Autogen Bioclear, Wiltshire, UK).

### 2.8 Statistical analysis

Data was analysed and significance between groups established using PrismGraph 3 software. Student's *t*-test and the Mann–Whitney test were used where appropriate. *p* < 0.05 was considered significant.

## 3 Results

### 3.1 Levels of ANA increase in hCR2^high^ mice with age

**Fig. 1:**
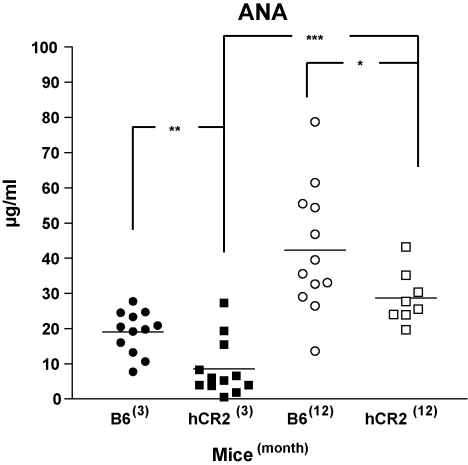
ANA levels increase in aged hCR2^high^ mice. Serum was collected from 12 C57Bl/6 (B6), B6.hCR2^high^ (hCR2) mice at 3 months or 12 months of age and applied to commercially available quantitative ANA ELISA following manufacturers instructions. Significance was calculated using an unpaired Student's *t*-test. ***p < 0.0005, **p < 0.005 and *p < 0.05.

Our recent analysis of B6^lpr^ mice (Pappworth et al., 2009) raised some questions regarding the long-term effects of hCR2 expression on B cell function. As a measure of B cell function during aging, we examined the levels of B cell dependent auto-nuclear antibodies in our original B6.hCR2^high^ mice using a commercially available quantitative ELISA. As expected, ANA levels increase significantly within the 6-month period between age of 3–12 months in normal B6 mice (42.2 ± 5.1 μg/ml or 2.2-fold) but remain about 50% lower than those seen in B6^lpr^ mice using this ELISA (Pappworth et al., 2009; Hayashi et al., 1989). A greater age dependent increase in ANA levels was observed in hCR2^high^ mice between 3 and 12 months of age (28.7 ± 2.6 μg/ml or 3.4-fold) relative to that found in B6 mice during aging. However, hCR2 transgenic mice always expressed lower ANA levels than age-matched B6 counterparts (average 32% lower, Fig. 1).

### 3.2 Increasing antigen dose improves Ig responses in hCR2 tg mice

**Fig. 2:**
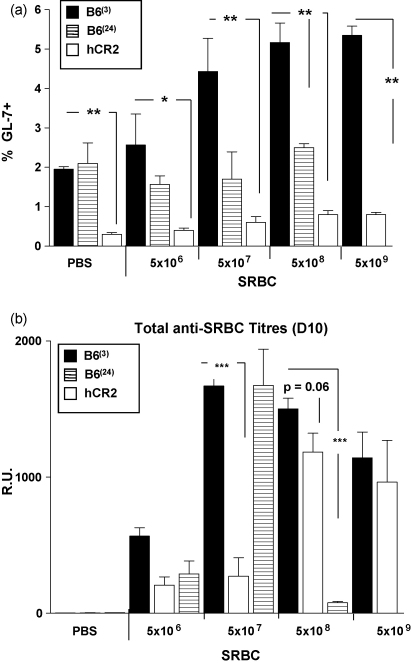
Increased antigen dose improves the immune response in hCR2^high^ mice. Mice were immunized with increasing concentration of SRBC in PBS. (a) After 10 days the germinal center reaction in the spleen was assessed by flow cytometry using forward and side scatter profiles as well as B220 expression to identify B cells and GL-7 to identify germinal center B cells. (b) Blood was collected at day 10, allowed to clot and anti-SRBC reactivity was then established by flow cytometry as described in Section 2. Groups of 2–6 C57Bl/6 (at 3 month (B6^(3)^) and 24 month (B6^(24)^)) and B6.hCR2^high^ (hCR2) mice were analysed. Mann–Whitney *U*-tests were used to establish significance. ***p < 0.0005, **p < 0.005 and *p < 0.05.

The analysis of ANA suggested that under certain conditions the unresponsive nature of hCR2^high^ mice might be reversed. In the case of both the C3^−/−^ and *Cr2*^−/−^ mice, increasing antigen dose or use of adjuvants resulted in partial recovery of the immune responses (Fischer et al., 1996; Wu et al., 2000). Equally, adjuvants have been used to improve Ig production in aged individuals and animal models with some success (Bates et al., 2008; Gahring and Weigle, 1990; Maletto et al., 2002; Sen et al., 2006; Szakal et al., 1990). Firstly, we investigated whether increasing antigen dose had any impact on the unresponsiveness of hCR2 tg and aged mice to SRBC. Regardless of the level of antigen used, germinal center responses as determined by measurement of the percentage of GL-7 positive B cells in the spleen, could not be returned to the levels noted in young hCR2 negative littermates. Rather, the proportion of GC B cells in hCR2^high^ mice remained around a third of the wild type littermate control values following immunization (Fig. 2a). Excessive Ag dose resulted in a minor recovery in the GC response, as the percentage of splenic GL-7^+^ B cells in 5 × 10^8–9^ SRBC immunized hCR2^high^ mice (0.6%) was double that seen in mice receiving PBS alone (*p* = 0.057), unlike the response to lower doses of Ag which remained relatively unchanged compared to the PBS control animals. No significant increase in splenic GL-7 levels was noted in immunized aged mice, irrespective of the dose of antigen used (Fig. 2a).

Surprisingly, hCR2 tg mice immunized with the highest antigen dose developed comparable anti-SRBC IgG responses, as measured at day 10, to that of similarly treated Wt littermates (Fig. 2b). Collectively, this data demonstrates that the defects in B cell maturation and function present in hCR2^high^ mice can be partially reversed by increasing Ag dose. The generation of anti-SRBC Ig by aged mice in response to increasing antigen dose was complex. Aged mice generated sub-optimal anti-SRBC Ig responses at low Ag dose (5 × 10^6^ SRBC) but comparable responses following moderate Ag dose (5 × 10^7^ SRBC), when compared with young B6 controls (Fig. 2a). However, aged mice receiving a high dose of SRBC failed to produce significant quantities of Ag-specific Ig suggesting these levels of antigen may be tolerizing in older animals.

### 3.3 Adjuvant does not reverse the phenotype in hCR2 tg mice

**Fig. 3:**
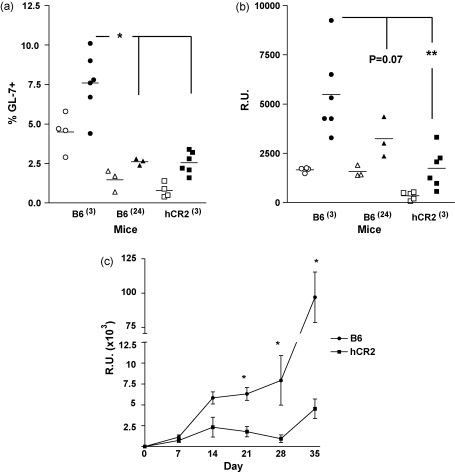
Adjuvant has little impact on the response to antigen in hCR2^high^ mice. Mice were immunized with 5 × 10^7^ SRBC/ml in complete Freund's adjuvant (filled symbols) or PBS (open symbols). A proportion of CFA treated animals were subsequently boosted at day 28 with 1 × 10^7^ SRBC/ml in incomplete Freund's adjuvant. (a) After 10 days the germinal center reaction in the spleen was assessed by flow cytometry using forward and side scatter profile as well as B220 expression to identify B cells and GL-7 to identify germinal center B cells, (b) sera was collected from mice at this time point and reactivity with SRBC established by flow cytometry as described in Section 2. (c) In a separate batch of mice, blood was collected at weekly intervals and allowed to clot, again anti-SRBC reactivity was then established by flow cytometry as described in Section 2. Groups of at least 3 C57Bl/6 either 3 or 24 months of age (B6^(3)^ and B6^(24)^, respectively) and 3-month-old B6.hCR2^high^ (hCR2^(3)^) mice were analysed. Mann–Whitney *U*-tests were used to establish significance. **p < 0.005 and *p < 0.05.

Considering the positive effect of increasing Ag dose on the immune response, we next assessed the effect of using adjuvant to drive the humoral response to a 5 × 10^7^ SRBC dose in the hCR2^high^ and aged mice. Initially, we analysed both the germinal center response and anti-SRBC response at day 10. Co-administration of adjuvant with Ag failed to fully normalize the B cell response in hCR2^high^ or aged mice, both in terms of GC B cells percentages and the production of anti-SRBC IgG (Fig. 3a and b). Responses in hCR2^high^ mice remained significantly lower than Wt controls. Aged animals showed a marginal improvement after addition of adjuvant but responses were low compared to young Wt mice. Indeed, the proportion of splenic GC B cells found in Ag immunized hCR2^high^ tg mice remained low even when compared with B6 animals injected with SRBC in absence of adjuvant. In short, adjuvant treatment did partially increase GL-7^+^ B cells and anti-SRBC levels in both hCR2 tg and aged mice when compared with hCR2^high^ or aged mice, injected with SRBC in the absence of adjuvant (Fig. 3a and b). In order to establish if CFA might eventually provide enough stimulus to overcome the initial defect in the hCR2^high^ mice, we next extended the analysis of the immune response to 35 days (including an additional boost) in a second experiment using IFA. Again, the immune response in hCR2^high^ mice failed and did not reach the levels noted in Wt mice. This was particularly evident after boost at day 28 (Fig. 3c), underlining a failure in GC and possibly memory cell formation in the hCR2^high^ mice. Thus, adjuvant also provides an increase in the relative response to Ag in hCR2^high^ and aged mice, but fails to reverse the intrinsic defects in B cell responsiveness displayed by both these groups when compared with Wt mice.

### 3.4 Aged hCR2^high^ responses to SRBC are equivalent to aged wild type mice

**Fig. 4:**
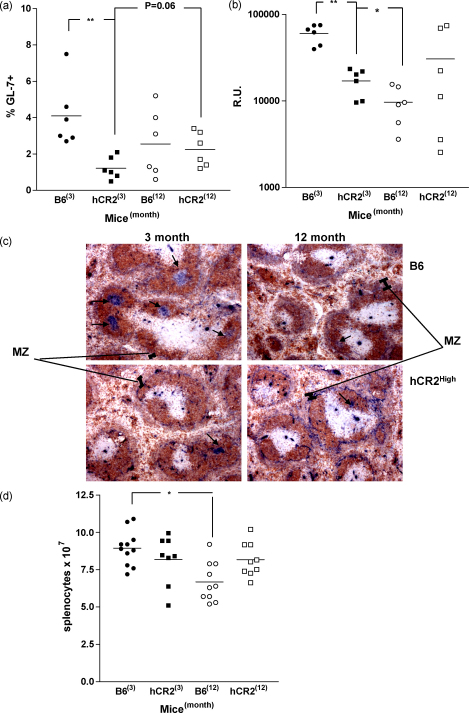
Wild type immune responses fail whilst hCR2^high^ responses improve with age. Three- and 12-month-old mice were immunized with 5 × 10^7^ SRBC/ml in PBS and after 10 days the spleen was snap frozen for immunohistochemistry or crushed and splenocytes counted prior to staining for flow cytometry. (a) Lymphocytes were identified using forward and side scatter profile as well as B220 expression to identify B cells and GL-7 to identify the germinal center B cells. 10,000 B cells were collected. (b) Blood was collected at day 10 by terminal exsanguination and allowed to clot. Anti-SRBC reactivity was then established by flow cytometry as described in Section 2. (c) Frozen sections of spleens from immunized mice were labeled with biotinylated PNA to label GCs (blue, identified by arrow) and counterstained with anti-IgD to label B cell follicles (brown) as described in Section 2. The “I” marks the approximate area of the marginal zone as defined by the PNA binding to marginal zone macrophages. Data shown is representative of random sampling across several sections of a spleen and from the mice analysed. (d) Total splenocytes, average of 3 counts on an improved Neubauer haemocytometer, are shown for naive or PBS only treated mice from each group as indicated. Groups of at least 6 C57Bl/6 (B6) and B6.hCR2^high^ (hCR2) mice were analysed. Mann–Whitney *U*-tests were used to establish significance. **p < 0.005 and *p < 0.05.

The marked recovery in Ag-specific Ig production observed in hCR2^high^ mice following high dose Ag administration (Fig. 2b) and elevated ANA levels in aged mice (Fig. 1) indicated that provision of sufficient Ag may overcome hCR2 dependent B cell defects. To explore whether the increase in responsiveness to auto-antigen in aged hCR2^high^ mice reflected persistent Ag stimulation or an age dependent increase in Ag responsiveness Wt and hCR2^high^ mice at either 3 or 12 months of age were injected with SRBC. Their germinal center and initial antibody response were then assayed. Ag injection of Wt mice (3-month old) induced a robust GC mediated anti-SRBC response (Fig. 4a–c) and as previously reported (Doria et al., 1978; Goidl et al., 1976; Kishimoto et al., 1976; Zharhary et al., 1977), the ability of B cells to generate specific Ig in response to Ag was significantly impaired in aged Wt mice. Importantly, the immune responsiveness noted in the hCR2^high^ mice did not deteriorate further than that seen at 3 months of age (Fig. 4a and b). In this respect it appears that hCR2^high^ mice at 3 months of age respond to foreign Ag in an equivalent manner to aged B6 mice (1–2 years old), i.e. their B cell immune response has been prematurely aged and this level of response neither declines or is significantly enhanced as hCR2^high^ mice age.

Immunohistochemistry analysis of immunized mice confirmed the GL-7 data (Fig. 4a), establishing that GC number and size were reduced in both hCR2^high^ mice and aged Wt mice when compared to the levels visible in young Wt mice. Interestingly, transgenic and older Wt mice with failed germinal center reactions were found to have similar splenic architecture, showing diffuse marginal zone (MZ) staining (PNA binding to marginal zone macrophages) (Fig. 4c). These data concurs with our earlier analysis of young hCR2^high^ mice (Birrell et al., 2005; Marchbank et al., 2002) and suggests that Wt mice adopt an similar MZ B cell population expansion and failed IgG production as they age. Thus, the defective B cell responsiveness of Ag challenged aged Wt and hCR2^high^ mice appears to be due to poor GC formation and refractory B cell maturation leading to disorganized splenic B cell and macrophage localization.

Total splenocyte numbers from unimmunized Wt and hCR2^high^ mice were also compared, to examine whether age dependent changes may contribute to the limited GC responses of aging mice. Examination of naive and PBS control animals during these experiments revealed that absolute splenocyte numbers were significantly reduced in older compared to younger B6 mice (Fig. 4d), despite their overall splenic weight being 33% greater with age (unpublished observations). When compared to young B6 mice, lower splenic numbers were also found in young and aged hCR2^high^ mice.

### 3.5 B cell populations are equivalent as hCR2^high^ mice age

In mice, splenic MZ B cells are a long-lived non-recirculating naive IgM^+^ B cell population which surround Ag-induced B cell follicles. This unique anatomical placement allows MZ B cells to screen for and contact blood-borne Ag inducing MZ B cell maturation and follicular migration (Pillai et al., 2005). Failure of this B cell maturation process, as found in young hCR2^high^ mice, leads to MZ B cell accumulation at the expense of follicular B cells (Birrell et al., 2005; Marchbank et al., 2002). We therefore next examined whether defective MZ B cell maturation (defined by MZ B cell accumulation) occurred in both aging Wt and hCR2^high^ mice as suggested by their impaired immune responsiveness (Fig. 4). When compared, the absolute numbers of splenic MZ B cells found in older Wt and hCR2^high^ mice was found to be almost identical (Table 1) and nearly double that found in young Wt mice indicating that MZ B cell populations expand with age as the relative rate of their maturation falls (in Wt mice). This raised the possibility that the reduction in the size of the MZ B cell pool in older hCR2^high^ mice might be driven by continued Ag contact during aging, such as those inducing ANA production. To examine this possibility, we also compared the rate of MZ B cell accumulation in aging hCR2^high^ mice in the absence of C3, as this is known to markedly reduce the acquired immune response to antigen (Fischer et al., 1996; Wessels et al., 1995). Interestingly, we found that C3 deficient animals also possessed an expanded MZ population, when compared to Wt animals of a similar age but loss of C3 reduced the extent of MZ changes in all animals during aging (Table 1). These data suggests that both signaling via C3 and presumably CR2 are important during the initial expansion and then the age-related alterations in the MZ B cell pool in both hCR2^high^ mice and Wt mice suggesting that older Wt and hCR2^high^ mice rely on similar B cell signaling pathways that lead to the continued maintenance of an enlarge MZ B cell pool.

### 3.6 Young hCR2^high^ mice have an immune system akin to aged mice

**Fig. 5:**
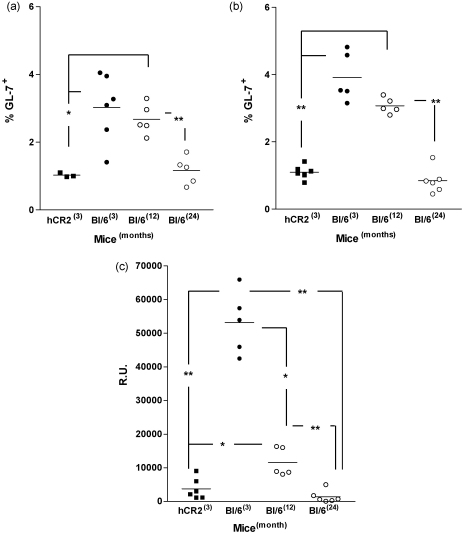
Germinal center and Ig response to SRBC fails in B6 over time B6 mice at 3, 12 and 24 months of age, as well as hCR2^high^ mice at 3 months age, were immunized with 5 × 10^7^ SRBC/ml in PBS or PBS as control and after 10 days the germinal center reaction in the spleen was assessed by flow cytometry using forward and side scatter profiles, B220 and GL-7 expression to identify the germinal center B cells. 10,000 B220^+^ events were collected flow cytometry. (a) Mice that have not been challenged with antigen, (b) mice challenged with SRBC and (c) blood was collected by terminal bleed at day 10 after antigen challenge and allowed to clot. Anti-SRBC titre was then established by flow cytometry as described in Section 2. Groups of between 3 and 6 mice were analysed. Mann–Whitney *U*-test were used to establish significance. **p < 0.005 and *p < 0.05.

**Fig. 6:**
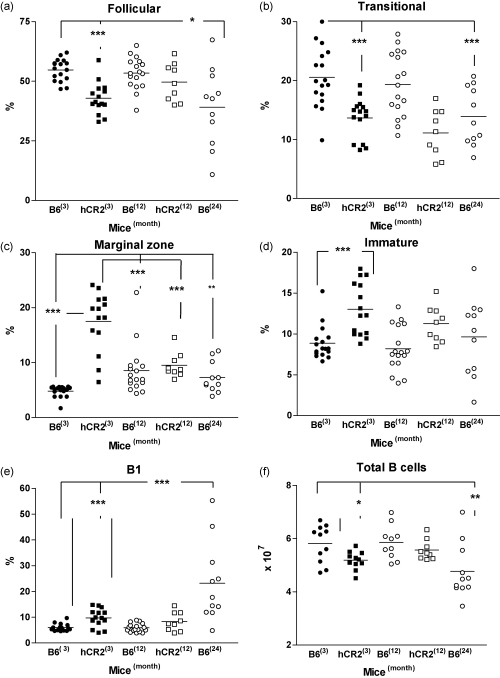
Young hCR2^high^ and aged B6 mice have equivalent changes in B cell sub-populations in the spleen. Splenocytes were isolated from B6 mice at 3, 12 and 24 months of age as well as from hCR2^high^ mice at 3 and 12 months of age. Cells were counted and Fc receptors blocked prior to being incubated with various fluorescent antibodies. 10,000 B220^+^ events were collected by flow cytometry and delineated according to the following surface marker profiles; (a) Follicular B cells (CD23^hi^, CD24^lo^, CD1d^−^), (b) transitional B cells (CD23^hi^, CD24^hi^, CD1d^int^), (c) marginal zone B cells (CD23^−^, CD24^lo^, CD1d^hi^), (d) immature B cells (CD23^−^, CD24^hi^, CD1d^int^) and (e) B1a B cells (B220^lo^, IgM^hi^, *CD5*^*+*^). Each sub-population is expressed as a percentage of total splenic B cells within that animal. (f) Shows total B cell numbers determined from the percentage of B220^+^ events collected in the lymphoid gate (negative for CD3e) in respect to the total splenocytes counted (mean of value of 3 counts using a haemocytometer) for each animal. At least 9 mice were used per group. Mann–Whitney *U*-test were used to establish significance. ***p < 0.0005, **p < 0.005 and *p < 0.05.

**Fig. 7:**
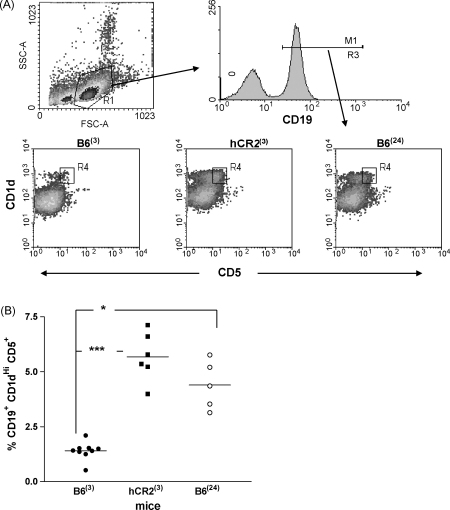
Young hCR2^high^ and aged B6 mice have significantly increased percentages of CD1d^hi^CD5^+^ B cells compared to young B6 mice. Splenocytes were isolated from B6 mice at 3 and 24 months of age as well as from hCR2^high^ mice at 3 months of age. Cells were counted and Fc receptors blocked prior to being incubated with various fluorescent antibodies. (a) Delineation of the B regulatory cell sub-population according to forward and side scatter and CD19^+^, CD1d^hi^ and CD5^+^ staining. (b) Percentage of CD1d^hi^ CD5^+^ determined from the individual mice as a percentage of CD19^+^ events collected in the lymphoid gate. 10,000 CD19^+^ events were collected by flow cytometry and at least 5 mice were used per group. Mann–Whitney *U*-test was used to establish significance. ***p < 0.0005 and *p < 0.05.

In previous experiments, we have shown that aging Wt and young hCR2^high^ mice may have similar defects in B cell driven immunity such as Ag responsiveness and B cell pool rearrangement. Therefore, we also compared the GC responses of aging Wt mice and young hCR2^high^ mice to SRBC (Fig. 5). The presence and extent of SRBC Ag-induced GC B cell formation in Wt mice was found to be highly age-dependent. In particular, GC B cell generation and the associated production of Ag-specific Ig completely fails by 2 years in B6 mice as is also found in 3-month-old hCR2^high^ mice (Fig. 5a and b). Analysis of B cell sub-populations in the spleen of naive mice also provides evidence that many of the changes seen in the aging mice are mirrored in the hCR2^high^ mice (Fig. 6) such as the apparent decline in the transitional B cell pool and the inverse expansion of the MZ and B1 B cell populations (Fig. 6b, c and e). Whether the reversal in MZ expansion continues in hCR2^high^ mice beyond 12 months is unclear as unfortunately no 2-year-old hCR2^high^ mice are available for such analysis. Total B cell numbers were also analysed and these largely correlated with the data gathered on absolute splenocytes numbers in these mice. A significant reduction in B cell numbers was found in the B6 mice at 24 months of age compared with 3-month-old B6 mice (Fig. 6f), whilst hCR2^high^ mice on the other hand, showed little change in their absolute B cell numbers as they aged (Fig. 6f and Table 1). Finally, we examined whether increased frequency of the newly described B10 B regulatory cell population (Breg) (Yanaba et al., 2008) might be contributing to the impaired B cell responsiveness of young hCR2^high^ and aged B6 mice. Surprisingly, analysis of the splenic B cell pool of 24-month-old B6 and young hCR2^high^ mice revealed a 3–5-fold increase in the percentage of CD1d^hi^CD5^+^ B cells relative to younger B6 counterparts (Fig. 7a and b). This unique finding suggests that the aging process within the immune system facilitates increased Breg accumulation and/or generation. The common expansion of a Breg population also further underlines the similarities seen in splenic B cell sub-population between aged B6 and hCR2^high^ mice as well as providing a potential explanation for the muted humoral immune response noted in both groups of mice.

## 4 Discussion

The B cell defect associated with expression of lambda light chain hCR2 transgene in the bone marrow compartment is likely a result of modified signaling at the point of heavy chain selection (Kulik et al., 2007; Twohig et al., 2007). We have previously found evidence of changes in internal signaling molecules and increased Ca^2+^ flux intensity, which is linked to decreased responses to endogenous and exogenous antigens. In short, mature B cells in hCR2^high^ mice showed evidence of increased negative selection, over-stimulation and a distinct anergic or irreversible unresponsive phenotype.

The data presented herein challenges the last conclusion and also suggests that a more detailed look at the existing data is warranted. Our recent study in B6.lpr mice indicated that whilst expression of hCR2 was protective in this background it could not totally prevent the accumulation of ANA and thus, may not prevent the onset of spontaneous autoimmune disease (Pappworth et al., 2009). Analysis of mice on the B6 background also suggested that much of the protective effect in terms of reduction in the levels of ANA was occurring in young animals and that with age, responses were returning to those seen in wild type animals. Indeed, given that young hCR2^high^ mice have approximately 60% fewer B cells than corresponding Wt mice (Marchbank et al., 2002), the lower level of ANA noted may simply represent a direct correlation with reduced B cell numbers in the hCR2^high^ mice, which does return towards normal as hCR2^high^ mice age (Fig. 6f). However, interpreting this data is difficult and complex because age is a potent modifier of the humoral immune response, shifting its focus from a strong coordinated response to foreign antigen in youth towards a less robust and increasingly distracted or clouded immune response to self, instead of foreign antigen, with age (Weksler et al., 1989). The examination of responses to SRBC in 1-year-old hCR2^high^ and hCR2 negative B6 mice demonstrated that immune responses were highly comparable at this time point (Fig. 4a and b). This was essentially a result of a marginally improving response in the hCR2^high^ mice and a clearly decreasing response in the hCR2 negative mice (Figs. 4 and 5). These data might be reconciled by the idea that T cell responses, and loss of T cell tolerance, may eventually drive the hCR2^high^ mice to either repopulate the B cell compartment with essentially autoreactive B cells or that the B1 B cell population in the hCR2^high^ mice continues to expand as seen in normal aging (Hu et al., 1993). Indeed, it is well known that the level of response to antigen in aged animals relies heavily on antigen type. Responses to TD antigens are markedly reduced with age whilst TI antigen responses are largely unaffected (Hu et al., 1993). Three-month-old hCR2^high^ mice do have a markedly reduced TD response (NP-KLH and SRBC (Birrell et al., 2005; Kulik et al., 2007; Marchbank et al., 2002)) but hCR2^high^ mice do not respond well to the T-independent antigen NP-Ficoll (Kulik et al., 2007) despite a marked increase in the MZ and B1a populations that are credited with responding to TI antigens. The data herein confirms that germinal center responses to the TD antigen SRBC are almost non-existent in 2-year-old wild type B6 mice and show clear defects at 1 year (Figs. 2a and 5). This data could be predicted from studies in other strains of mice carried out in the 1970s, which relied on lack of class switch and affinity maturation to infer defects in the germinal center responses (Doria et al., 1978; Goidl et al., 1976; Kishimoto et al., 1976; Zharhary et al., 1977) and more recent data, which showed an almost complete absence of the somatic hypermutation process in aged C57BL/6 mice (Miller and Kelsoe, 1995). Indeed, it has been previously reported that increasing antigen dose increases Ab forming cell number and Ig titre but germinal center formation/function remains impaired in aged animals. Furthermore, this type of improvement has been noted in aging mice following use of CpG, Flaggelin and CFA as adjuvants (Bates et al., 2008; Gahring and Weigle, 1990; Maletto et al., 2002; Sen et al., 2006; Szakal et al., 1990). In all cases, the use of adjuvant markedly increased the Ig titre without a marked improvement in GC formation/function. Taken in combination with our own data, these findings further underline the similarity between the immune response parameters in aged and hCR2^high^ mice.

Equally, the changes in B cell population in wild type mice as they age (Fig. 6) is also consistent with earlier studies and illustrate that as B6 mice age they also expand predominantly the B1a and also to some degree the MZ B cell populations at the expense of B2 (follicular) and the transitional B cell populations. The marked expansion of CD1d^hi^CD5^+^ B cells, which was recently shown to be a potent Breg cell population (Bouaziz et al., 2008; Matsushita et al., 2008; Yanaba et al., 2008) offers a possible explanation for the apparent reduction in immune responses in both hCR2^high^ and aged B cell populations. These Breg cells may be expanded in aged mice in an effort to quell the rise of autoreactive B cells and this might equally be the case in the hCR2^high^ mice, as paradoxically increased expression of hCR2 was expected to result in B cells with increased reactivity, not the muted responses we found (Kulik et al., 2007). The presence of an expanded Breg population in hCR2^high^ mice also fits with our data showing that B cells from hCR2^high^ mice respond normally when stimulated *ex vivo* but are unresponsive *in vitro* (Kulik et al., 2007). Coincidently, the existence of an expanded Breg population in the hCR2^high^ mice would likely have been previously added to the MZ or B1 subsets, depending on the original analysis criteria, and again could partly explain why the MZ expansion did not translate to increase immune reactivity to TI antigens (Kulik et al., 2007). Notably, the level of MZ B cell expansion in the 3-month-old hCR2^high^ mice is much more pronounced than that seen in the aged mice, although there is evidence of MZ changes in aging B6 mice as visualized by immunohistochemistry (Fig. 4c) and by cell sub-population analysis (Fig. 6c). This data could reflect a direct role for CR2 in the function/formation of the MZ B cell population, as has been previously suggested (Srivastava et al., 2005), that is not replicated in the aging animals as levels of endogenous mCR2 are not significantly changed over time (unpublished observation).

A reduction in total B cell numbers is clearly demonstrated in hCR2^high^ mice in the periphery of 3-month-old mice, yet a B cell reduction is not generally associated with the ageing immune system (Marchbank et al., 2002). However, we found that total B cell numbers were significantly lower in B6 mice at 24 months of age and absolute splenocytes numbers were decreasing in the B6 mice at 12 months of age (numbers in the hCR2^high^ mice also decrease in real terms, considering that overall splenic weight has increased by 33% yet cell numbers remain static over that time). There are documented reductions in the B2 B cells in aged animals but these are generally negated by large expansions in the B1 cells (Hu et al., 1993) and although this is visible in the B6 mice, the level of expansion is clearly not sufficient to fully bridge the gap. The overall number of B cells in hCR2^high^ mice at 3 months of age is significantly lower than aged matched B6 mice. However, hCR2^high^ mice appear to maintain their splenocytes and B cell numbers better over time (Table 1, Figs. 4d and 6f). The reduction in B2 B cells in 3-month-old hCR2^high^ mice is compensated by a small increase in B1a B cell numbers as well as an increase in IgM titres (B1a cells are thought to be the main producers of this isotype of antibody). Thus, the presence of hCR2 appears to aid B1a B cell function (and at the expense of B2 B cells). This data fits with the theory that B1a cells rely on constant BCR signaling to maintain their numbers, hence additional signaling from hCR2 or in the case of aging through constant interaction with self leads to expansion of this B cell population.

Numbers of pre-B cells and then numbers of immature and transitional B cells have been shown to be reduced in aged mice (Johnson et al., 2002a; Kline et al., 1999; Sherwood et al., 2000), reductions which are largely mirrored in the young hCR2^high^ mice (Marchbank et al., 2002). We have established that apoptosis levels are increased in hCR2^high^ mice prior to or immediately at the point of sIgM expression (Pappworth et al., 2009), suggesting that the switch from Pre-B cell to immature B cell is the critical step in both aging mice and hCR2^high^ mice. Interestingly, the alteration in bone marrow B cells in aged mice has been associated with a reduction in both mRNA and protein levels of the B cell survival molecule BCL-X_L_ (Kirman et al., 1998; Sherwood et al., 2003), and raises the possibility that hCR2 could be disrupting the production of this B cell survival molecule. Support for this idea comes from our data in CD19 KO mice, as CD19 was found to aid B cell survival in hCR2 transgenic mice (Twohig et al., 2007) and suggests that a focused examination of BCR/co-receptor signaling associated proteins at this time point in the hCR2 transgenic mice is likely to yield useful data.

Currently, there is little data on the effects of aging on BCR engagement and its down stream signaling events. However, studies in aging or senescent T cells may provide some clues to understanding what is happening in both hCR2^high^ and wild type B cells during the aging process. Evidence from ‘exhausted’ T cell clones, such that you may find in chronic diseases and aging suggest that changes in T cell susceptibility to apoptosis do occur (Koch et al., 2006). Our own data suggests that hCR2^high^ mice do have B cells with significantly different apoptotic profiles compared with wild type mice (Pappworth et al., 2009), suggesting a further similarity with the aged immune system. Again, studies of T cell receptor signaling in aged cells has demonstrated many examples of changes in the down stream signal transduction pathways, including alterations in tyrosine kinase activity, intracellular free calcium, inositol phosphates and protein kinase C (PKC) (Fulop, 1994; Fulop et al., 1999; Ghosh and Miller, 1995; Miller, 2000; Pawelec et al., 2001). Our analysis of B cell receptor signaling, whether in combination with or absence of CR2 cross-linking, has illustrated clear alteration in tyrosine phosphorylation patterns and propagation of intracellular calcium in the hCR2^high^ mice (Kulik et al., 2007). T cell anergy was originally described as a lack of co-stimulation during the initial T cell receptor engagement with antigen and is highly important in the maintenance of tolerance in the periphery (Nel, 2002; Nel and Slaughter, 2002). The mechanisms that induce T cell anergy are largely unknown but impaired Ras activation, as part of the defective TCR-mediated signaling along the PKC–Ras–MAPK pathways, seem to play a pivotal role in maintenance of the anergic state in primary T cells (Gajewski et al., 1994). Interestingly, ageing naïve T cells and memory T cells display a proliferative unresponsiveness as a result of alterations in these same signal transduction pathways, with the PKC–Ras–MAPK pathway being severely impaired with ageing (Miller, 2000; Pawelec et al., 2001). Thus, the unresponsive phenotype displayed by hCR2^high^ mice, which we previously determined as not classical anergy, may well be a result of chronic stimulation though CR2/BCR, which resembles the situation normally reached by aged B cells. Additionally, the generation or accumulation of increased numbers of the CD1d^hi^CD5^+^ Breg cells in both aging and hCR2^high^ animals may also arise due to increased or chronic signaling through BCR/CR2. Thus, it is likely to be highly informative to examine the ontogeny of Breg populations in various complement deficient strains over time.

Overall, the data assembled in this manuscript and over the last decade suggests that the defects associated with premature expression of the co-signaling molecule CR2 on the B cell surface during B cell development leads to fundamental changes in B cell signaling which have hallmarks of an unresponsive and aged B cell. Please refer to Table 2 to see a summary of the similarities found between aged and hCR2^high^ mice. Exactly how similar B cells from young mice expressing hCR2 and B cells isolated from 2-year-old mice or from classical anergy models is an interesting and challenging question which we hope to investigate through array and proteomics approaches in due course.

## Figures and Tables

**Table 1:** Changes in B cell populations over time.

	3 months		12 months	
	B cell	MZ	B cell	MZ
B6	58.2 ± 0.21	2.8 ± 0.14	58.6 ± 0.2	5.0 ± 0.63***
hCR2^high^	51.9 ± 0.1	9.8 ± 0.76	55.7 ± 0.13**	5.0 ± 1.11***
C3^−/−^	56.7 ± 0.9	6.1 ± 0.05	58.3 ± 0.67	7.3 ± 0.43
C3^−/−^hCR2^high^	53.4 ± 0.1	9.4 ± 1.09	59.0 ± 0.43**	8.3 ± 0.55

Flow cytometry was carried out on splenocytes as described in Section 2. Shown are the B cell (B220^+^ CD19^+^) and marginal zone (MZ) B cell (B220^+^CD1d^hi^CD24^lo^) numbers (×10^6^ ± SEM) as calculated from % cells in the lymphoid gate and total splenic counts. At least 6 mice were included per group and Mann–Whitney *U*-test was used to establish *p* values.

** *p* < 0.001 compared with same population at the earlier time point.

*** *p* < 0.0005 compared with same population at the earlier time point.

**Table 2:** Comparison of aged B6 and young hCR2^hi^.

	Aged B6	hCR2^hi^
GC response	Highly impaired response	Highly impaired response
Ag–low dose	Highly impaired response	Highly impaired response
Ag–high dose	Normal response	Impaired response
Ag–very High dose	Impaired response	Impaired/Normal response
Ag + adjuvant	Impaired/Normal response	Impaired/Normal response
Splenocytes/B cells	Reduced	Reduced
Follicular B cells	Reduced	Reduced
Transitional B cells	Reduced	Reduced
B1 B cells	Highly expanded	Expanded
Marginal zone B cells	Expanded & disorganized	Highly expanded & disorganized
Bregs (CD1d^hi^CD5^+^)	Expanded	Expanded

Details listed in this table are based on C57Bl/6 mice greater than 12 months of age (aged B6) or hCR2^hi^ mice (at least 3 months of age and not greater than 4 months of age) when compared directly to the humoral immune responses or B cell populations measured in young C57Bl/6 (3-month-old Wt mice).
